# Supplementary material for: Extracellular c-di-GMP Plays a Role in Biofilm Formation and Dispersion of Campylobacter jejuni
Source: Microorganisms. 2022 Oct 14;10(10):2030. doi: 10.3390/microorganisms10102030 (PMC9608569; doi:10.3390/microorganisms10102030)
Supplement: Supplementary file 1 [file microorganisms-10-02030-s001.zip › microorganisms-1955918-supplementary.pdf]

Extracellular c-di-GMP plays a role in biofilm formation and dispersion of *Campylobacter jejuni*

Bassam A. Elgamoudi[1], Kirstie Starr[1] and Victoria Korolik[1ξ]

[1] Institute for Glycomics, Griffith University, Gold Coast campus, QLD 4222, Australia.

[ξ]Corresponding author, Victoria Korolik, Institute for Glycomics, Griffith University, Gold Coast campus, QLD 4222, Australia, email v.korolik@griffith.edu.au

Table S1. Primer sets were used in this study.

Primer Name	Primer sequence 5′ to 3′	Restriction Site
gyrA- F	CCACTGGTGGTGAAGAAAATTTA	N/A
gyrA- R	AGCATTTTACCTTGTGTGCTTAC	N/A
Tlp8- F	GAATACCATGGCGGTAATTGAAT	N/A
Tlp8- R	CCTTGATCTCTGAAAGGGAGTAT	N/A
Tlp6- F	AGCTGCACGCGCAGGTGAACATG	N/A
(Cj0448c)Tlp6-R	TCAACTTCTGTGGTTGCTTTTTG	N/A
(Cj0284c)CheA-F	ATCATGGATGTGGTTTTAGAGTC	N/A
CheA-R	GGTTCTATATCCATACCAATAGC	N/A
Cj0643-F (cbrR)	TCAATGCAAGTTTCACTTTC	N/A
Cj0643-R	CTATTGTTCTTACGGCTAGT	N/A
luxS gene (Cj1198-F)	GGCTTCATCTAAAGAATGCATTG	N/A
luxS gene (Cj1198-R)	AGCCATGAAAGATGTTTTAAGCG	N/A

Table S2. Effect of ex-c-di-GMP on biofilm for *C. jejuni* strains, *C. jejuni* 11168-O (11168), *C. jejuni* 81-176 (81-176), and *C. jejuni* 81116 (81116). Each data point is presented as the mean± standard errors of three independent experiments. The asterisk (*) indicates a statistically significant difference compared to the untreated control (*p<0.05, **p<0.007, ***p<0.0001).

**Test strains**	**Total biofilm ($OD_{590\ nm}$)**					**Biofilm inhibition (%)**			
	**ex-c-di-GMP concetration (μM)**								
	untreated	50	100	200	400	50	100	200	400
11168-O	0.83±0.05	0.58±0.04***	0.52±0.1***	0.42±0.04***	0.38±0.1***	29.04	37.35	49.4	53.1
81-176	1.33±0.3	1.06±0.02	1.01±0.05*	0.83±0.08**	0.63±0.1**	20.3	24.06	37.6	52.6
81116	1.47±0.2	1.25±0.1*	1.19±0.05*	1.01±0.04**	0.8±0.01***	15.1	19.3	30.4	44.85

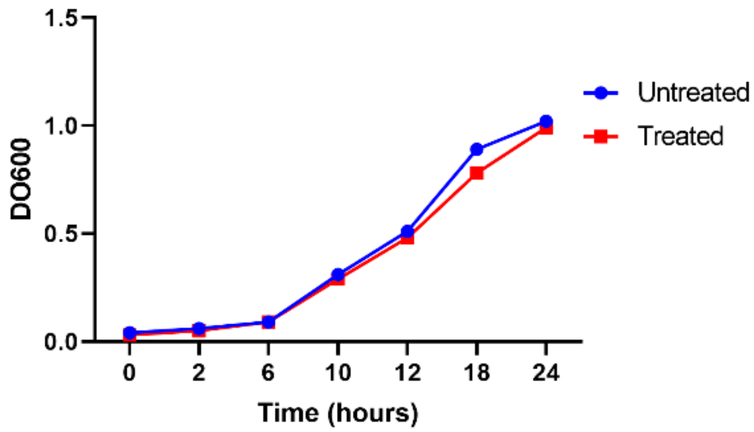

Figure S1. The growth rate of *C. jejuni* with and without 200 µM ex-c-di-GMP.

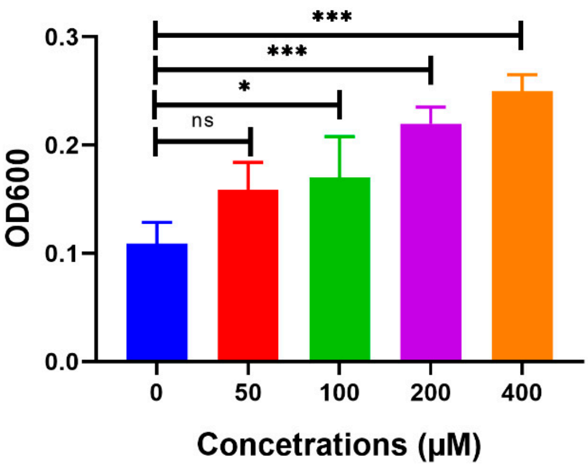

Figure S2. Release of planktonic cells during dispersion of *C. jejuni* 11168-O biofilms in the presence of ex-c-di-GMP at different concentrations. Ex-c-di-GMP at different concentrations induced dispersion of the existing biofilm and increased the number of planktonic cells (dispersed cells). The asterisk (*) indicates a statistically significant difference compared to the untreated control($p<0.05$).

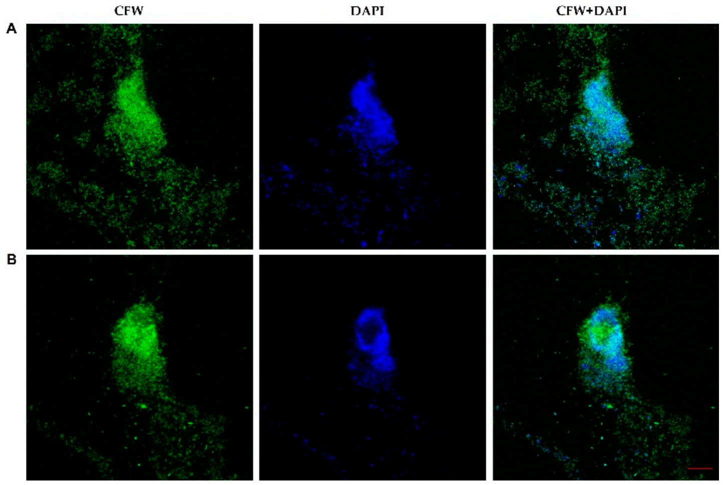

Figure S3. CLSM images of biofilm disperion associated with ex-c-di-GMP. (A) *C. jejuni* 11168-O biofilm (48 hours). (B) After 3 hrs incubation with 200 μM ex-c-di-GMP (Scale bar = 10μm). Calcofluor White (CFW) (green, polysaccharides) and DAPI (blue, DNA). Red arrows indicate the void formation indicative of dispersion.
